# Supplementary material for: Catalytically efficient Ni-NiOx-Y2O3 interface for medium temperature water-gas shift reaction
Source: Nat Commun. 2022 May 4;13:2443. doi: 10.1038/s41467-022-30138-5 (PMC9068818; doi:10.1038/s41467-022-30138-5)
Supplement: Supplementary file 1 — Supplementary Information [file 41467_2022_30138_MOESM1_ESM.pdf]

Supplementary Information for

Catalytically Efficient Ni-NiO$_x$-Y$_2$O$_3$ Interface for Medium Temperature Water-Gas Shift Reaction

Kai Xu[1], Chao Ma[2], Han Yan[1], Hao Gu[3], Wei-Wei Wang[1], Shan-Qing Li[4], Qing-Lu Meng[1], Wei-Peng Shao[1], Guo-Heng Ding[1], Feng Ryan Wang[3]*, Chun-Jiang Jia[1]*

[1]Key Laboratory for Colloid and Interface Chemistry, Key Laboratory of Special Aggregated Materials, School of Chemistry and Chemical Engineering, Shandong University, Jinan 250100, China.
[2]College of Materials Science and Engineering, Hunan University, Changsha 410082, China.
[3]Department of Chemical Engineering, University College London, Roberts Building, Torrington Place, London WC1E 7JE, UK.
[4]Key Laboratory of Micro-Nano Powder and Advanced Energy Materials of Anhui Higher Education Institutes, Chizhou University, Chizhou, 247000, China.

*Corresponding author. Email: ryan.wang@ucl.ac.uk, jiacj@sdu.edu.cn.

Table of Contents

Supplementary Figures

Supplementary Tables

Supplementary Reference

Supplementary Figures:

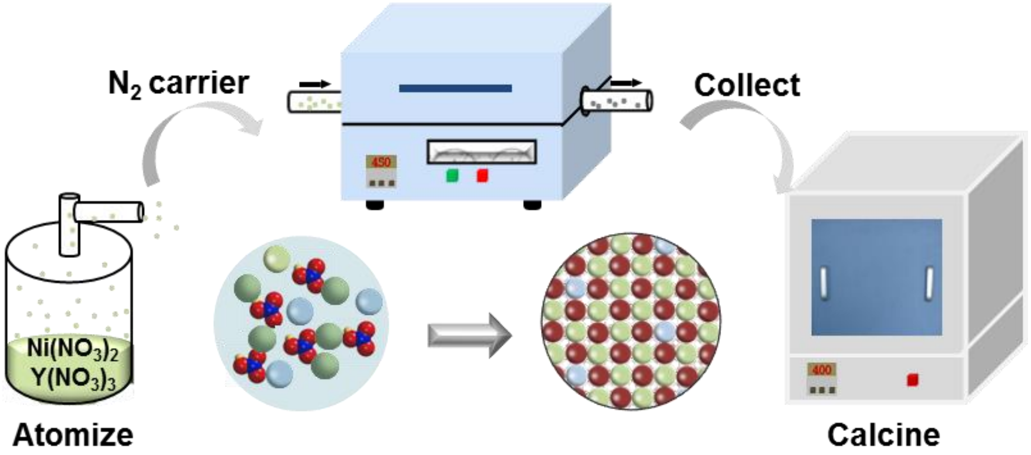

Supplementary Fig. 1 | Synthesis of catalysts. Schematic illustration on the stepwise formation of $Ni_aY_bO_x$ catalysts.

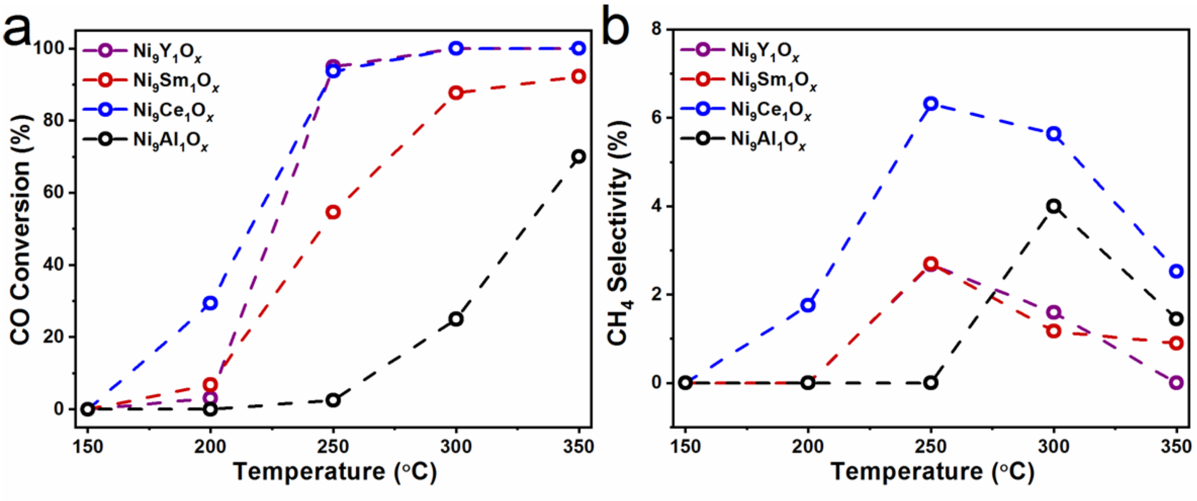

Supplementary Fig. 2 | Catalytic performance of $Ni_9M_1O_x$. (a) Temperature-dependent activity test of a series of $Ni_9M_1O_x$ catalysts ($Ni_9Y_1O_x$, $Ni_9Sm_1O_x$, $Ni_9Ce_1O_x$ and $Ni_9Al_1O_x$); (b) the CH_4 selectivity of a series of $Ni_9M_1O_x$ catalysts ($Ni_9Y_1O_x$, $Ni_9Sm_1O_x$, $Ni_9Ce_1O_x$ and $Ni_9Al_1O_x$); the reaction gas content was 2%CO, 10%H_2O, and the equilibrium gas was N_2, GHSV = 42,000 $cm^3\ g_{cat}^{-1}\ h^{-1}$.

Supplementary Fig. 2 showed the CO conversion and CH_4 selectivity for $Ni_9M_1O_x$ catalysts. The WGS activity of $Ni_9Y_1O_x$ was similar to that of $Ni_9Ce_1O_x$, and higher than that of $Ni_9Sm_1O_x$ and $Ni_9Al_1O_x$. Besides, for CH_4 selectivity, $Ni_9Y_1O_x$ was lower than $Ni_9Ce_1O_x$. In general, the catalysts prepared with the participation of Y_2O_3 showed excellent catalytic performance.

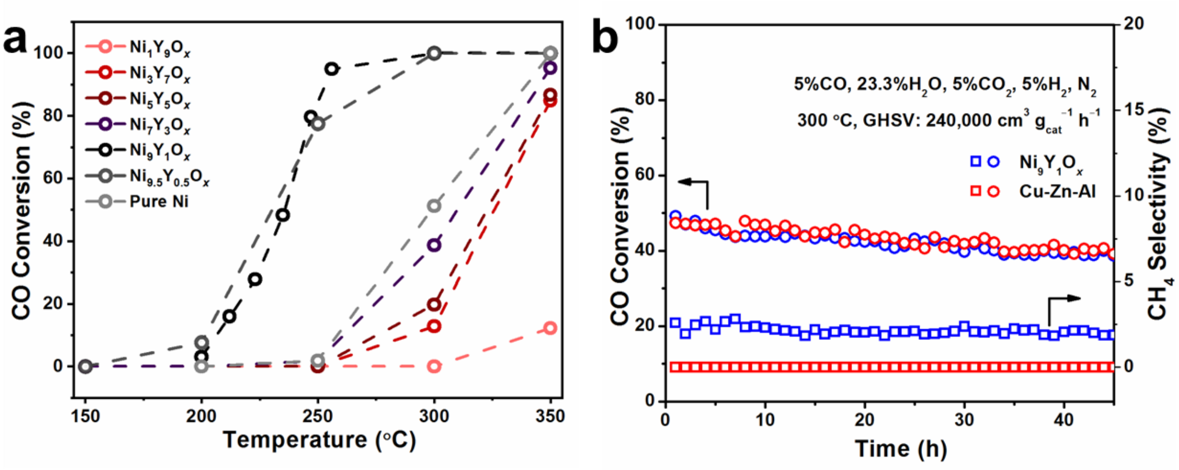

Supplementary Fig. 3 | Catalytic performance of $Ni_9Y_1O_x$. (a) Temperature-dependent activity test of the catalysts with different Ni/Y ratios; the reaction gas content was 2%CO, 10%H_2O, and the equilibrium gas was N_2, GHSV = 42,000 cm^3 g_{cat}^{-1} h^{-1}; (b) time-on-stream tests of the $Ni_9Y_1O_x$ and commercial Cu-Zn-Al in the stream of 5% CO, 23.3% H_2O, 10% H_2, 5% CO_2, N_2.

Supplementary Fig. 3a showed the WGS reaction activities of the $Ni_aY_bO_x$ catalysts with different Ni/Y ratios. With increasing the Ni content, the catalytic activity increased gradually. The $Ni_9Y_1O_x$ catalyst exhibited the best activity. Further increase of the Ni/Y ratio to 9.5:0.5 led to a less active catalyst, and the activity of pure Ni sample was even worse.

Supplementary Fig. 3b showed the stability test in the stream of 5% CO, 23.3% H_2O, 10% H_2, 5% CO_2, N_2. The $Ni_9Y_1O_x$ catalyst and the commercial Cu-Zn-Al had similar CO conversion in this stream. It also maintained good stability at high temperature and with an ultra-high GHSV, in which the conversion decayed from 47% to 40% in 45 h.

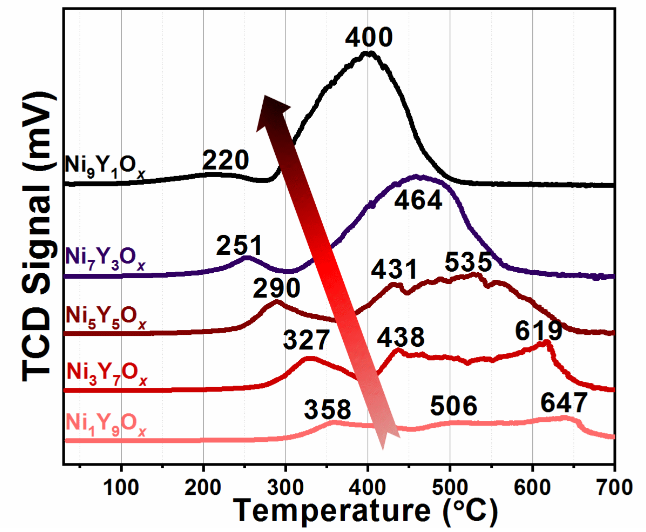

Supplementary Fig. 4 | H_2-TPR profile of the fresh $Ni_aY_bO_x$ catalysts.

With the increase of Ni content, the reduction peaks gradually shifted to low temperature, indicating that more Ni content was beneficial to the reduction of catalyst. Compared to the reduction peaks of pure NiO and pure Y_2O_3 prepared by the same method (Supplementary Fig. 5a), the different reduction peaks of $Ni_aY_bO_x$ catalysts were considered as step-by-step reduction of Ni species at different locations. Overall, the reduction peaks of pure NiO and $Ni_aY_bO_x$ located differently, which manifested a certain interaction between NiO and Y_2O_3. In addition, for the $Ni_9Y_1O_x$ catalyst, we found that only a small part of the surface NiO related to the Ni-Y_2O_3 interface was reduced to Ni before 300 °C, while a large amount of NiO was reduced mainly at 400 °C. This was also consistent with the phase change observed by *in situ* XRD result (Fig. 4a).

Besides, we quantitatively calculated the theoretical and actual hydrogen consumption of the catalysts (Supplementary Table 2). The results showed that the actual hydrogen consumption of all samples was higher than the theoretical value. And the higher values became smaller as the Y content decreased. These phenomena indicated that the redox property of Y_2O_3 was also increased due to the formation of the interface, which confirmed the interaction existed in the Ni-NiO_x-Y_2O_3 interface.

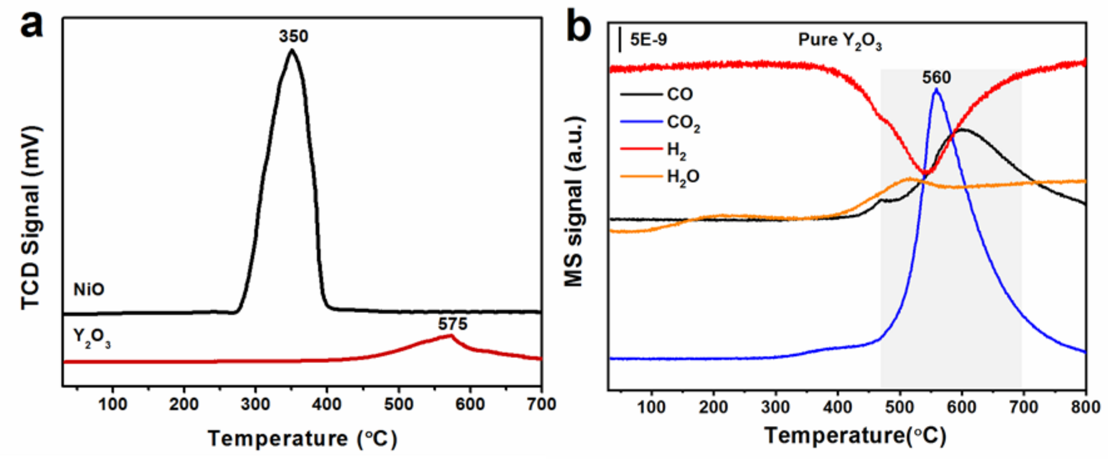

Supplementary Fig. 5 | H_2-TPR profile of pure oxide. (a) H_2-TPR profile of pure NiO and Y_2O_3 with a TCD detector; (b) H_2-TPR profile of pure Y_2O_3 sample with a mass spectrometer.

Notably, the reduction peak of pure Y_2O_3 at 575 °C was found to be the reduction of surface carbonate species, rather than the intrinsic reduction of Y_2O_3 (Supplementary Fig. 5b).

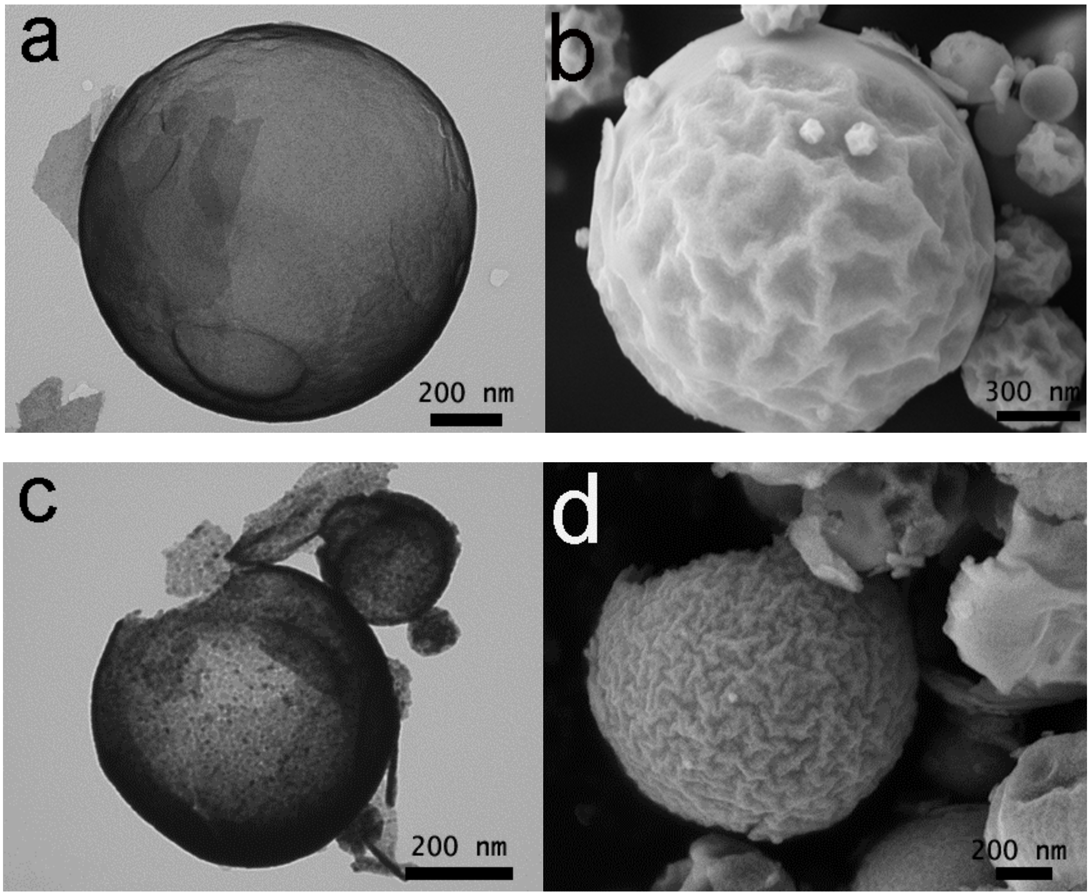

Supplementary Fig. 6 | Transmission electron microscope (TEM) images of the $Ni_9Y_1O_x$ catalyst. (a) before catalysis test and (c) after catalysis test; scanning electron microscope (SEM) images of the $Ni_9Y_1O_x$ catalyst: (b) before catalysis test and (d) after catalysis test.

For the $Ni_9Y_1O_x$ catalyst, each component was uniformly dispersed before the reaction, and there were no large particles. After the catalytic test (Supplementary Fig. 6c), the catalysts still maintained spherical morphology, indicating that the structure of the catalyst was stable and there was no obvious sintering. However, we found that particles with size of 8−15 nm on the surface $Ni_9Y_1O_x$-used were formed.

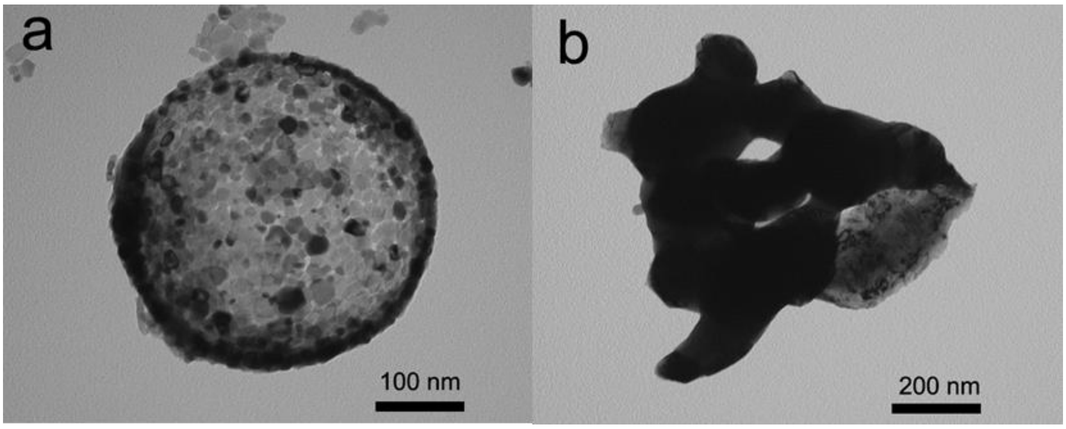

Supplementary Fig. 7 | Transmission electron microscope (TEM) images of pure Ni sample. (a) Fresh sample; (b) used sample.

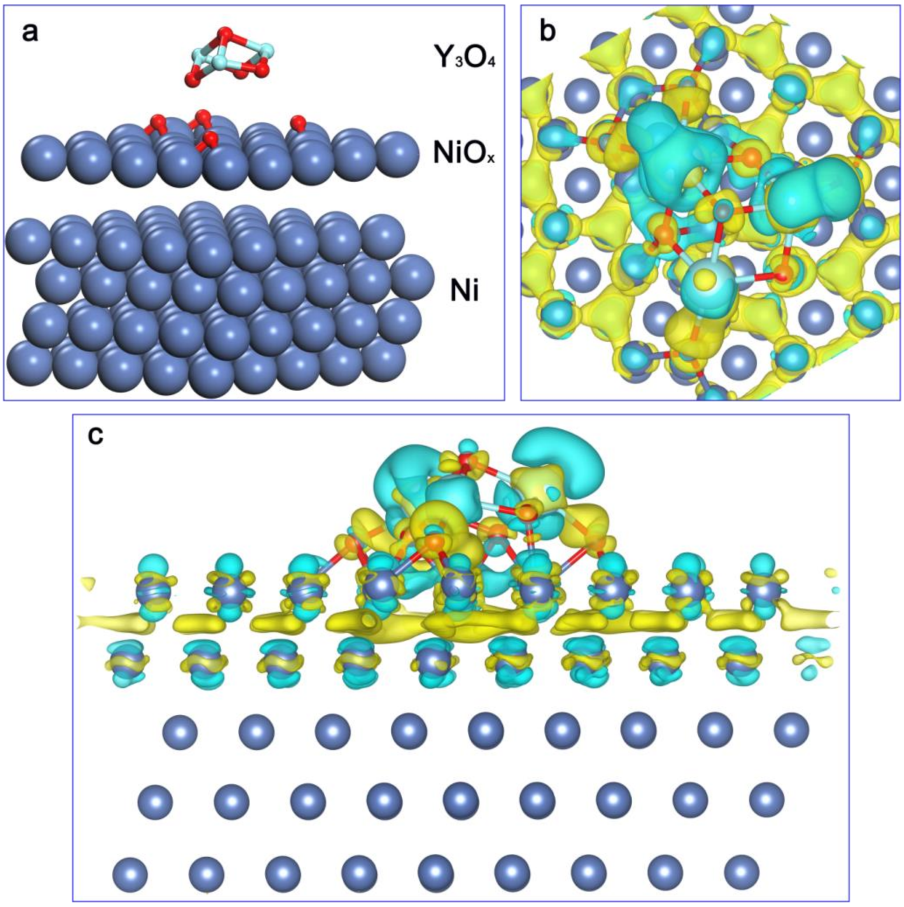

Supplementary Fig. 8 | The structural diagram of the Y_3O_4/NiO_x/Ni{111} model. (a) exploded view; (b) (c) deformation charge density diagram (using VESTA[1] software), the yellow and cyan represent the density of positive charge increases and decreases, respectively.

The top Ni-layer was separated from Ni{111} and formed NiO_x-layer by adding four oxygen atoms. Three Y atoms along with four oxygen atoms formed the Y_3O_4 layer. The deformation charge density diagram showed that the borderline between Ni{111} and NiO_x was an accumulation area for electron. The Bader charge analysis suggested that 0.187 negative charge transferred from Ni{111} to NiO_x, i.e., there was no obvious gain and loss of electrons. Based these two aspects, we stood for the metallic bond. As regards Y_3O_4, 1.643 negative charges transferred to NiO_x, and the bonding ways were Ni-O and Y-O. So, the chemical interaction between Y_3O_4 and NiO_x were the ionic bond.

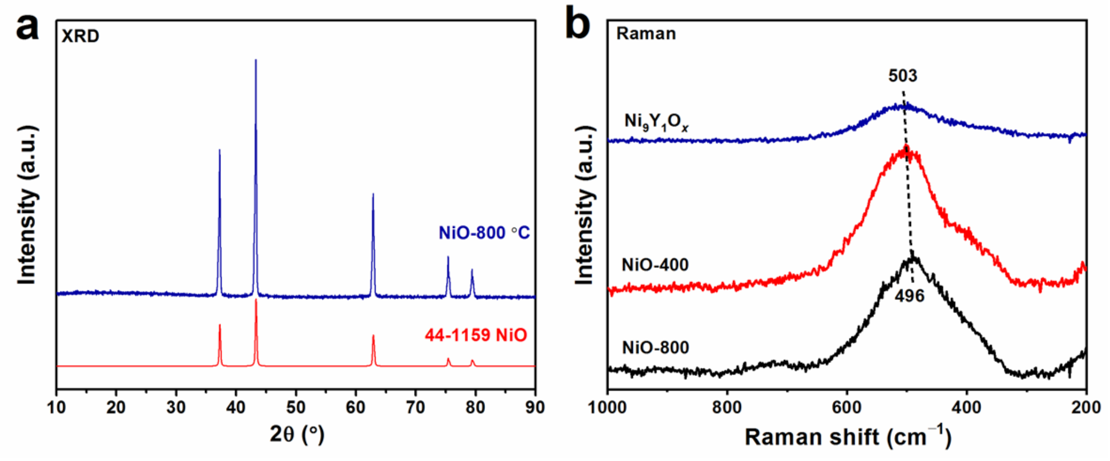

Supplementary Fig. 9 | Characterization of NiO. (a) XRD pattern of NiO after calcination at 800 °C. (b) Raman spectra of the samples of $Ni_9Y_1O_x$, NiO-400 and NiO-800.

In order to verify that the vibration peak at about ~505 cm^{-1} was related to NiO, we further conducted the XRD and Raman measurements of the NiO samples prepared by the same method after calcination at 800 °C. All the samples showed the same peak position in Raman spectra, so this broad peak was a characteristic peak for NiO.

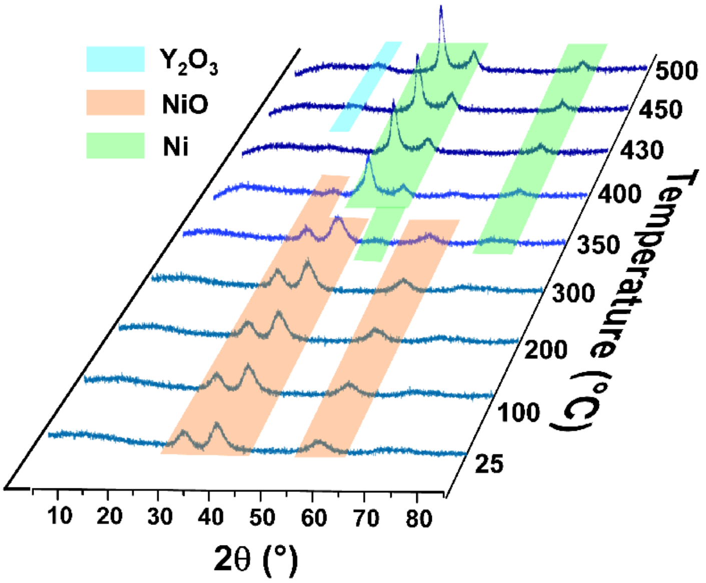

Supplementary Fig. 10 | *In situ* XRD patterns under 2% CO/Ar for the $Ni_9Y_1O_x$ catalyst.

The *in situ* XRD patterns under CO atmosphere demonstrated that there was mainly NiO phase from 25 to 350 °C. And the diffraction peak of metallic Ni appeared from 350 °C, and NiO was reduced to Ni completely at 400 °C. This was consistent with the *in situ* Raman test result (Fig. 4c).

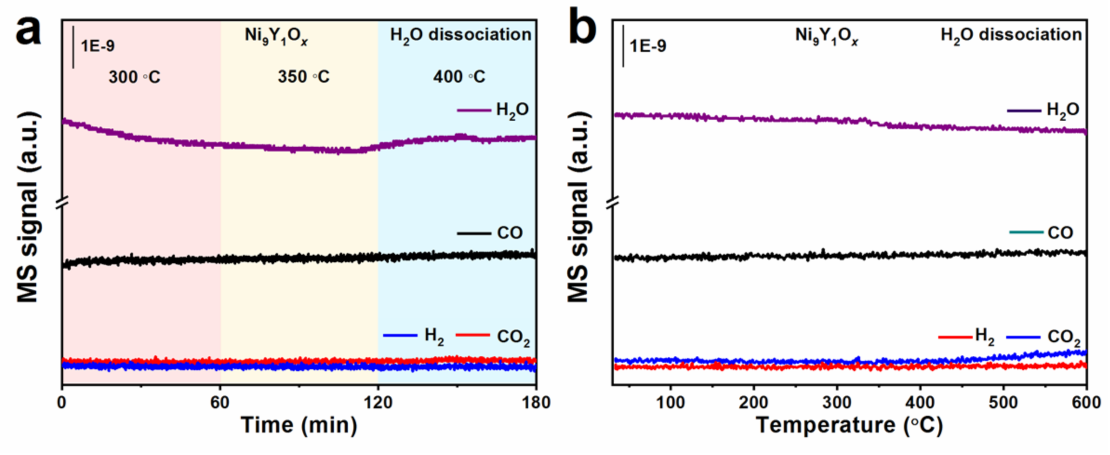

Supplementary Fig. 11 | The H_2O dissociation experiment of $Ni_9Y_1O_x$. (a) The H_2O dissociation experiment of $Ni_9Y_1O_x$ at different temperatures (300, 350 and 400 ºC); (b) the H_2O dissociation experiment of $Ni_9Y_1O_x$ with a heating process from 25 to 600 ºC.

For the redox mechanism, CO reacted with the surface oxygen species to form CO_2 and vacancies, where H_2O dissociated to form H_2. Following this mechanism, H_2 production could be observed during the H_2O dissociated process. However, there was no H_2 signal in either the thermostatic test or the temperature-programmed test, indicating that the reaction path did not follow the redox mechanism.

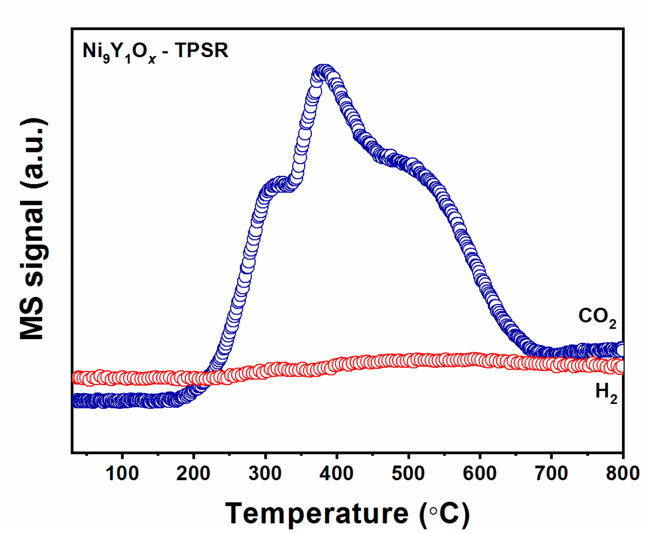

Supplementary Fig. 12 | The temperature-programmed surface reaction (TPSR). TPSR on the $Ni_9Y_1O_x$ pretreated by ~3%H_2O/Ar, then under 2%CO/Ar atmosphere during the heating process.

We verified the associative mechanism through the TPSR experiment. However, the results of the temperature-programmed experiment showed a wide range of desorption signals of CO_2, which was caused by the strong adsorption capacity of the catalyst for CO.

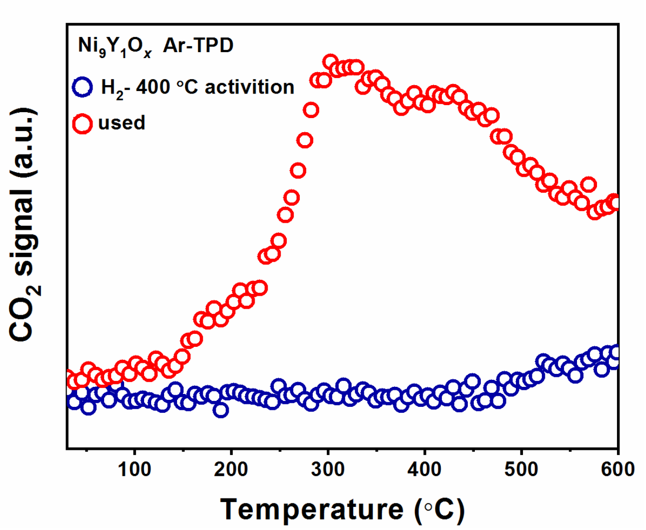

Supplementary Fig. 13 | Ar-TPD results of $Ni_9Y_1O_x$. The CO_2 signal of temperature-programmed desorption (TPD) using Ar as the carrier gas for the $Ni_9Y_1O_x$-used sample and the H_2-400 ºC activation sample.

In order to further verify the strong adsorption capacity of the catalyst for CO, the TPD experiments using Ar as the carrier gas were carried out. After the WGS reaction, an obvious CO_2 desorption signal was observed for the used sample, due to the presence of CO as the reactant. In contrast, there was almost no CO_2 desorption for H_2 pretreated samples. This result was consistent with the TPSR (Supplementary Fig. 12) result.

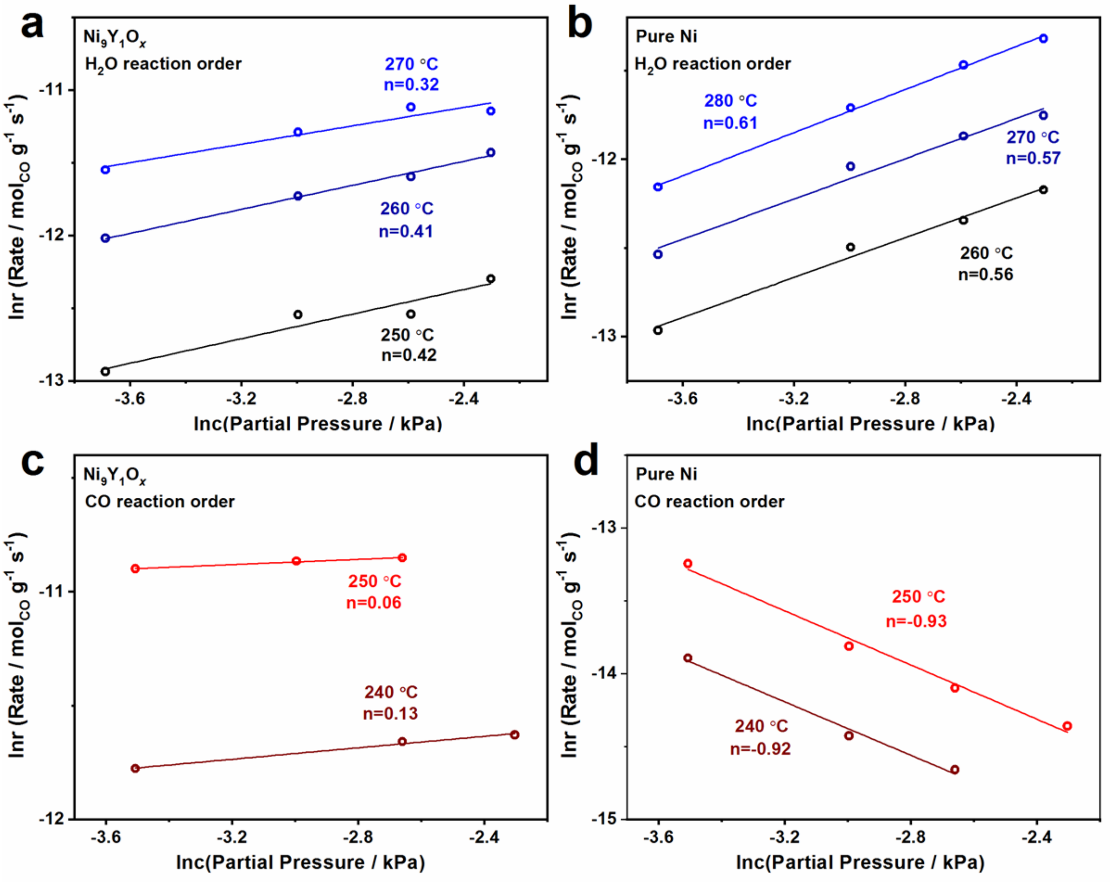

Supplementary Fig. 14 | The results of reaction orders. (a, b) Reaction orders of the H_2O for $Ni_9Y_1O_x$ (250 ºC, 260 ºC, 270 ºC), and pure Ni sample (260 ºC, 270 ºC, 280 ºC), respectively; (c, d) reaction orders of the CO for $Ni_9Y_1O_x$ (240 ºC, 250 ºC) and pure Ni sample (240 ºC, 250 ºC), respectively.

The H_2O reaction order of the pure Ni sample (0.57 at 270 ºC) was larger than that of the $Ni_9Y_1O_x$ sample (0.32 at 270 ºC), indicating that the latter was easier to adsorb and dissociate H_2O. The CO reaction order of the $Ni_9Y_1O_x$ catalyst closed to 0 indicated that the reaction was little affected by the CO concentration, because of the strong CO adsorption capacity. Besides, for pure Ni sample, the CO reaction order was negative, which might due to the CO poisoning.

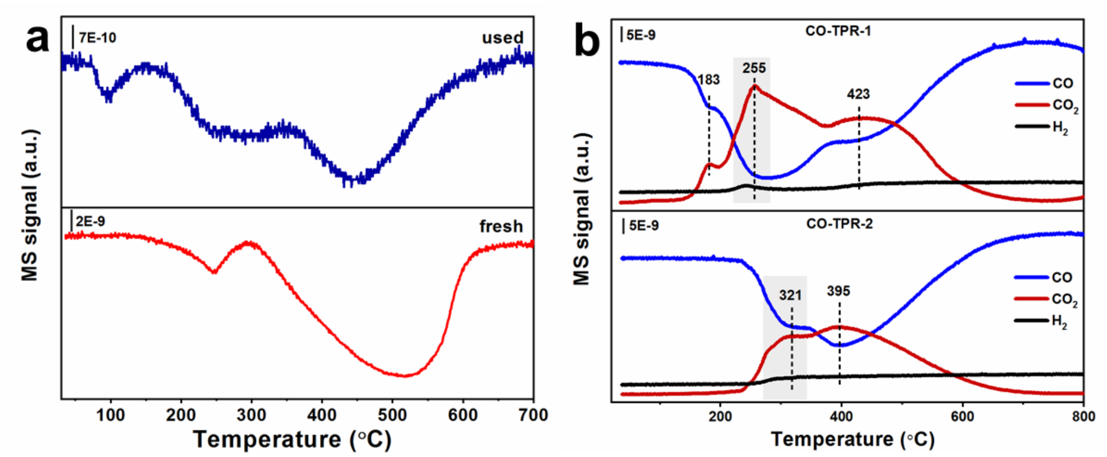

Supplementary Fig. 15 | TPR of $Ni_9Y_1O_x$ catalyst. (a) H_2-TPR-MS experiment of the fresh and used samples; (b) cyclic CO-TPR experiments for CO consumed and CO_2 evolved against temperature for $Ni_9Y_1O_x$ catalyst (Between CO-TPR-1 and CO-TPR-2, the catalyst was treated in ~3%H_2O/Ar atmosphere at 300 ºC).

The result of Supplementary Fig. 15a showed that after the reaction, the used sample was reduced by H_2 again, the consumption peak still appeared. The reduction peak was considered as the reduction of NiO_x species formed in the reaction atmosphere. As shown in Supplementary Fig. 15b, the catalyst was treated in ~3%H_2O/Ar atmosphere at 300 ºC between CO-TPR-1 and CO-TPR-2. Same as Fig. 5b, there were mainly three reduction peaks in the CO-TPR-1. The one at lower temperature (183 °C) was the reduction of small-size NiO, and the one at higher temperature (423 °C) should be the reduction of large-size NiO. The reduction peak at 255 °C was accompanied by the generation of H_2, which resulted from the reaction between CO and hydroxyl species. For CO-TPR-2, two main reduction peaks could be observed, among which the higher temperature reduction peak was considered as the reduction of NiO_x species formed in the reaction atmosphere. Accordingly, the reduction peak at 321 °C was also accompanied by the generation of H_2 signal, indicating the generation of surface hydroxyl was recyclable.

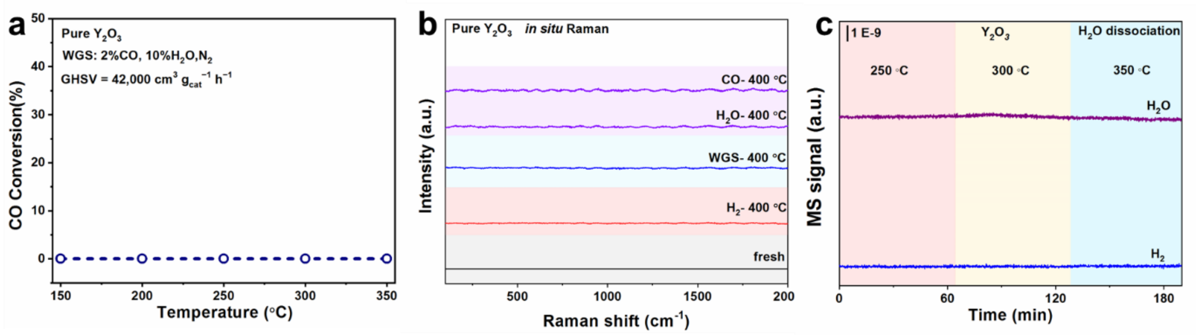

Supplementary Fig. 16 | Characterization of Y_2O_3. (a) The CO conversion of pure Y_2O_3 for the WGS reaction; (b) *in situ* Raman spectra of pure Y_2O_3 in different streams; (c) the H_2O dissociation experiment of pure Y_2O_3 at different temperatures (250, 300 and 350 ºC).

In order to ensure that pure Y_2O_3 did not contribute to the dissociation of H_2O, activity test, *in situ* Raman and H_2O dissociation experiments were carried out for pure Y_2O_3 samples. Firstly, from the activity test results, pure Y_2O_3 has no catalytic activity for the WGS reaction (Supplementary Fig. 16a). Secondly, the *in situ* Raman of pure Y_2O_3 did not show any valuable information (Supplementary Fig. 16b). It showed that Y_2O_3 basically did not have any obvious signal due to poor crystallization, and no significant changes after switched to H_2, WGS reaction stream, H_2O and CO atmosphere. We could not directly observe the dissociation of H_2O. So we further verified it using H_2O dissociation experiments (Supplementary Fig. 16c). There was no H_2 signal was observed in the range of 250−350 °C in ~3H_2O/Ar stream, which indicated that pure Y_2O_3 could not effectively dissociated H_2O.

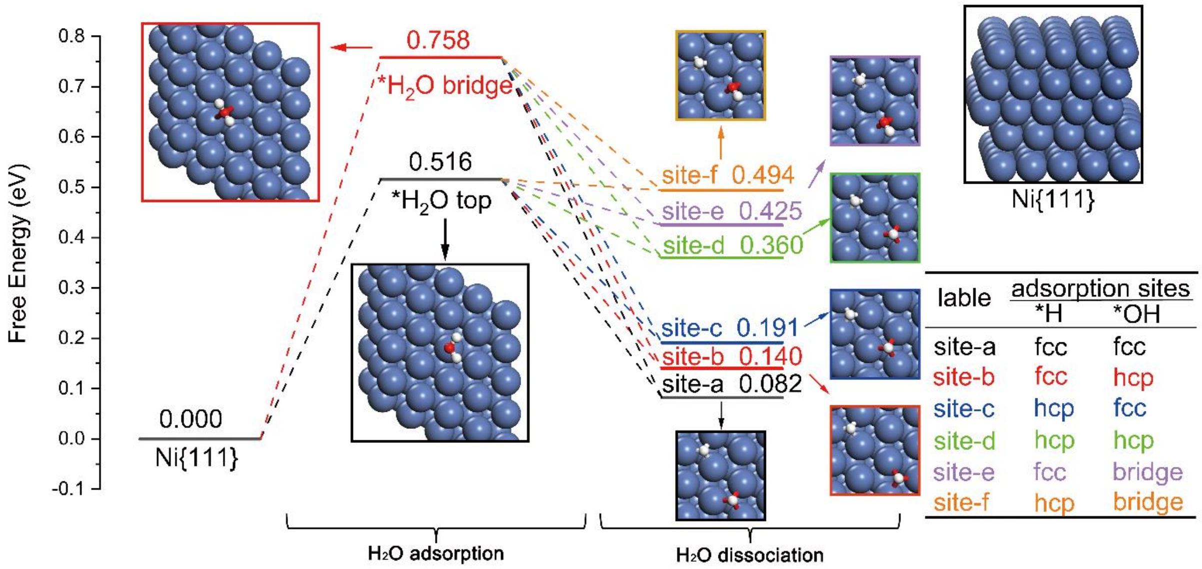

Supplementary Fig. 17 | Structures for various steps of H_2O activation at Ni{111}. The two H_2O adsorption sites (top and bridge) and six H_2O dissociation sites (site a to f) were simulated at 300 ºC, and the Gibbs free energies (Δ*G*) of the WGS reaction at air inlet, i.e., partial pressure of H_2O was 10 kPa. The structures of intermediates were attached near to the numbers, and the character of structure was listed in the table in the lower-right corner.

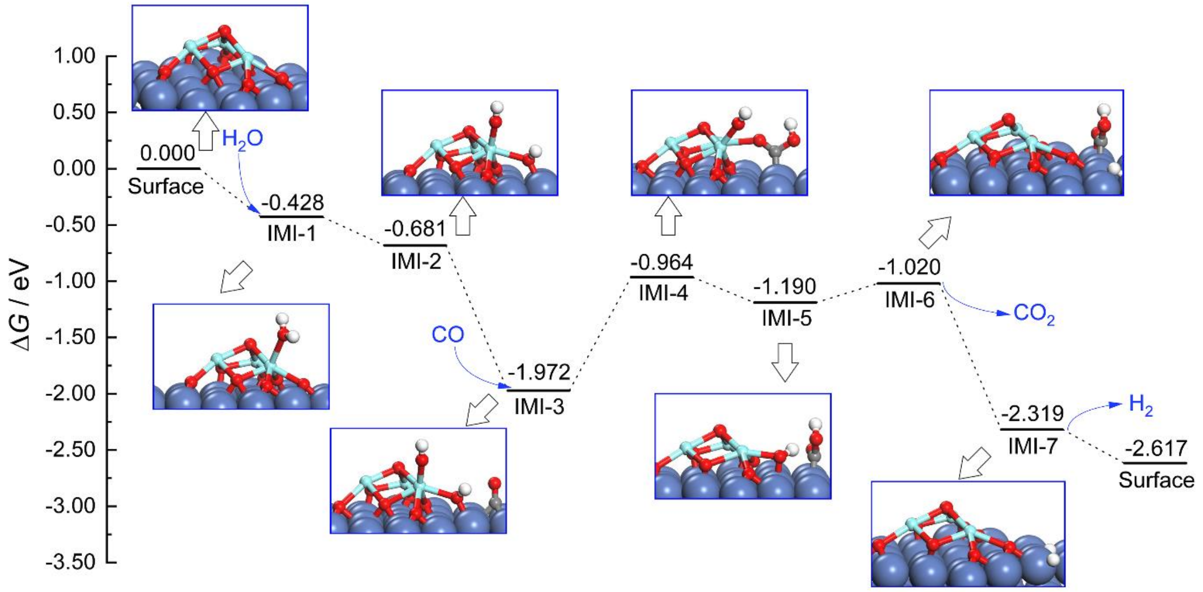

Supplementary Fig. 18 | The Gibbs free energy changes at the inlet of reactor. The reaction temperature was 300 °C, and the partial pressures of CO, H_2O, CO_2 and H_2 were 2000, 10000, 1 and 1 Pa respectively.

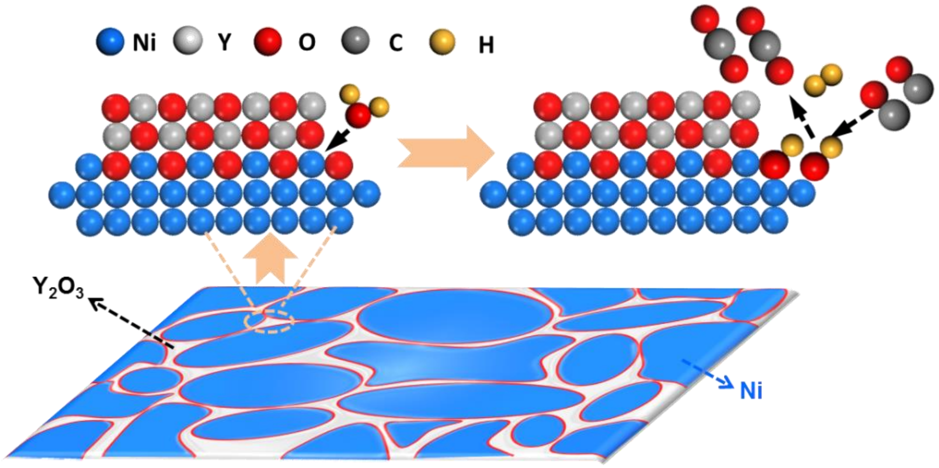

Supplementary Fig. 19 | Role of the Ni-NiO$_x$-Y_2O_3 interfaces in catalyzing the WGS reaction in the $Ni_9Y_1O_x$ catalyst.

Supplementary Tables

Supplementary Table 1 Comparison of catalytic performances for WGS reaction over various catalysts.

Catalyst	Reaction conditions	Reaction temperature (°C)	Reaction rate ($\mu mol_{CO}\ g_{cat}^{-1}\ s^{-1}$)	TOF (s^{-1})	Ref.
**$Ni_9Y_1O_x$**	**2% CO, 10% H_2O, balance N_2**	**250**	**37.2**	**0.37**	**This work**
		**300**	**140.6**	**1.39**	
		**350**	**434.7**	**4.31**	
1Re-10Ni/CeO_2	5% CO, 10% H_2O, balance He	300	30.0	–	2
Ni_{20}/$CeLaO_x$	10% CO, 20 % H_2O, balance He	300	35.2	–	3
		350	36.3	–	
Cu_4Ni_{16}/$CeLaO_x$	10 % CO, 20 % H_2O, balance He	300	31.1	–	
		350	35.9	–	
Ni@TiO_{2-x} (450)	6% CO, 24% H_2O, 70% Ar	350	356.8	3.8	4
Inverse CeO_2/Cu	2% CO, 10% H_2O, balance N_2	300	47.5	0.058	5
Cu-Ce(La)O_x	2% CO, 10% H_2O, balance N_2	300	9.0	–	6
$Cu_{0.3}Fe_{0.7}O_x$	2% CO, 10% H_2O, balance N_2	300	12.2	–	7
Ir_1/FeO_x	2% CO, 10% H_2O, balance He	300	43.4	2.31	8
5Pt/0.06Na-TiO_2	3% CO, 10% H_2O, balance He	250	38.4	1.58	9
2% Au/α-MoC	10.5% CO, 21% H_2O, 20%N_2, balance Ar	200	325	–	10
0.2Pt-Mo_2N	2% CO, 10% H_2O, balance N_2	300	232	–	11

Supplementary Table 2 The Ni contents, S_{BET} and the H_2 consumption of the $Ni_aY_bO_x$ catalysts.

Sample	Mole ratio of Ni/Y	Ni content (wt %)[a]	NiO content (wt %)[b]	S_{BET} (m^2 g^{-1})[c]	H_2 consumption (μmol g_{cat}^{-1})	
					Actual[d]	Theoretic
$Ni_9Y_1O_x$	9:1	67.3	85.6	55.5	11974	11413
$Ni_7Y_3O_x$	7:3	47.7	60.7	27.8	11319	8093
$Ni_5Y_5O_x$	5:5	31.3	39.8	25.2	9384	5307
$Ni_3Y_7O_x$	3:7	17.4	22.1	22.2	7410	2947
$Ni_1Y_9O_x$	1:9	5.4	6.8	12.9	3522	907

[a] The theoretic percentage of metallic Ni in the total weight.

[b] The theoretic percentage of NiO in the total weight.

[c] Determined with N_2 adsorption.

[d] The actual date was determined with H_2-TPR profile (Supplementary Fig. 3).

Supplementary Table 3 The value of binding energy for the XPS fitting result.

<table>
<tr><th>Sample</th><th colspan="3">Element</th><th>Binding Energy (eV)</th></tr>
<tr><td rowspan="4">$Ni_9Y_1O_x$-fresh</td><td rowspan="2">Ni</td><td rowspan="2">Ni^{2+}</td><td>$2p_{3/2}$</td><td>853.84, 855.43</td></tr>
<tr><td>$2p_{1/2}$</td><td>871.15, 872.85</td></tr>
<tr><td rowspan="2">Y</td><td rowspan="2">Y^{3+}</td><td>$3d_{5/2}$</td><td>156.68, 157.94</td></tr>
<tr><td>$3d_{3/2}$</td><td>158.72, 159.99</td></tr>
<tr><td rowspan="6">$Ni_9Y_1O_x$-used</td><td rowspan="4">Ni</td><td rowspan="2">Ni^{2+}</td><td>$2p_{3/2}$</td><td>855.55</td></tr>
<tr><td>$2p_{1/2}$</td><td>873.04</td></tr>
<tr><td rowspan="2">Ni^0</td><td>$2p_{3/2}$</td><td>852.70</td></tr>
<tr><td>$2p_{1/2}$</td><td>869.81</td></tr>
<tr><td rowspan="2">Y</td><td rowspan="2">Y^{3+}</td><td>$3d_{5/2}$</td><td>157.98</td></tr>
<tr><td>$3d_{3/2}$</td><td>160.11</td></tr>
<tr><td rowspan="4">$Ni_9Y_1O_x$-used (quasi in situ XPS)</td><td rowspan="2">Ni</td><td rowspan="2">Ni^0</td><td>$2p_{3/2}$</td><td>853.22</td></tr>
<tr><td>$2p_{1/2}$</td><td>870.50</td></tr>
<tr><td rowspan="2">Y</td><td rowspan="2">Y^{3+}</td><td>$3d_{5/2}$</td><td>158.30</td></tr>
<tr><td>$3d_{3/2}$</td><td>160.38</td></tr>
</table>

Supplementary Table 4 The fitting results of XAFS analysis.

Samples	Y-O			Y-Y			ΔE_0(eV)
	R (Å)	C.N.	σ^2 (Å^2)	R (Å)	C.N.	σ^2 (Å^2)	
$Ni_9Y_1O_x$-fresh	2.357 ± 0.071	7.671 ± 2.033	0.016 ± 0.005	—	—	—	−1.319 ± 2.676
$Ni_9Y_1O_x$-used	2.343 ± 0.056	5.155 ± 0.392	0.008 ± 0	3.613 ± 0.098	1.182 ± 2.014	0.002 ± 0.015	−0.511 ± 2.065

R: distance; C.N.: coordination number; σ^2: Debye-Waller factor;
ΔE_0: correction to the photoelectron energy origin.

Supplementary Table 5 The change of particle size during the *in situ* XRD in 5% H_2/Ar.

Reaction Temperature (°C)	species	particle size (nm)[a]	species	particle size (nm)[a]
25	NiO	3.2	—	—
100	NiO	2.9	—	—
200	NiO	3.3	—	—
300	NiO	2.8	—	—
350	Ni	4.8	—	—
400	Ni	5.5	Y_2O_3	2.9
430	Ni	6.2	Y_2O_3	3.4
450	Ni	6.8	Y_2O_3	4.2
500	Ni	9.6	Y_2O_3	5.3

[a] Determined by the XRD patterns and Scherrer formula

Supplementary Table 6 The partial pressures of CO, H_2O, CO_2 and H_2 at air outlet.

Temperature (℃)	partial pressures (Pa)			
	CO	H_2O	CO_2	H_2
150	2000	10000	1	1
200	1938	9938	61	61
212	1680	9680	319	319
223	1444	9444	555	555
235	1034	9034	966	966
247	408	8408	1592	1592
256	101	8101	1899	1899
300	1	8000	2000	2000
350	1	8000	2000	2000

* One Pa in the table means the substance is not detected.

Supplementary Table 7 Cumulative changes in the Gibbs free energies (Δ*G*) of the WGS reaction at air outlet (The partial pressures of CO, H_2O, CO_2 and H_2 are listed in Supplementary Table 6).

Temperature (℃)	Δ*G* (eV)							
	IMI-1	IMI-2	IMI-3	IMI-4	IMI-5	IMI-6	IMI-7	Surface
150	-0.521	-0.723	-2.282	-1.279	-1.519	-1.241	-2.031	-1.835
200	-0.489	-0.707	-2.173	-1.168	-1.404	-1.158	-2.256	-2.054
212	-0.481	-0.703	-2.141	-1.136	-1.370	-1.133	-2.163	-1.927
223	-0.473	-0.698	-2.111	-1.105	-1.338	-1.109	-2.115	-1.886
235	-0.463	-0.693	-2.069	-1.063	-1.295	-1.074	-2.054	-1.834
247	-0.453	-0.687	-1.998	-0.992	-1.223	-1.011	-1.966	-1.756
256	-0.445	-0.682	-1.913	-0.905	-1.136	-0.932	-1.876	-1.681
300	-0.417	-0.670	-1.584	-0.576	-0.803	-0.633	-1.555	-1.476
350	-0.385	-0.659	-1.455	-0.444	-0.666	-0.540	-1.432	-1.487

Supplementary Table 8 Cumulative changes in the Gibbs free energies (ΔG) of the WGS reaction at air inlet, i.e., partial pressures of CO, H_2O, CO_2 and H_2 are 2000, 10000, 1 and 1 Pa respectively.

Temperature (℃)	ΔG (eV)							
	IMI-1	IMI-2	IMI-3	IMI-4	IMI-5	IMI-6	IMI-7	Surface
150	-0.521	-0.723	-2.282	-1.279	-1.519	-1.241	-2.031	-1.835
200	-0.490	-0.707	-2.175	-1.170	-1.406	-1.160	-2.426	-2.393
212	-0.482	-0.704	-2.150	-1.144	-1.379	-1.141	-2.413	-2.419
223	-0.475	-0.701	-2.127	-1.121	-1.355	-1.125	-2.402	-2.444
235	-0.468	-0.698	-2.102	-1.096	-1.329	-1.108	-2.390	-2.471
247	-0.460	-0.694	-2.078	-1.071	-1.303	-1.091	-2.377	-2.498
256	-0.455	-0.692	-2.060	-1.052	-1.282	-1.078	-2.367	-2.519
300	-0.428	-0.681	-1.972	-0.964	-1.190	-1.020	-2.319	-2.617
350	-0.398	-0.671	-1.876	-0.866	-1.088	-0.961	-2.263	-2.727

Supplementary Reference

1. Momma, K. and Izumi, F. VESTA 3 for three-dimensional visualization of crystal, volumetric and morphology data. *J. Appl. Crystallogr.* **44,** 1272–1276 (2011).
2. Chayakul, K., Srithanratana T. and Hengrasmee S. Catalytic activities of Re–Ni/CeO_2 bimetallic catalysts for water gas shift reaction. *Catal.Today* **175,** 420–429 (2011).
3. Lin, J. et al. Hydrogen production by water–gas shift reaction over bimetallic Cu–Ni catalysts supported on La-doped mesoporous ceria. *Appl. Catal. A: Gen.* **387,** 87–94 (2010).
4. Xu, M. et al. TiO_{2-x}-modified Ni nanocatalyst with tunable metal–support interaction for water–gas shift reaction. *ACS Catal.* **7,** 7600–7609 (2017).
5. Yan, H. et al. Construction of stabilized bulk-nano interfaces for highly promoted inverse CeO_2/Cu catalyst. *Nat. Commun.* **10,** 3470 (2019).
6. Li, Y., Fu, Q. & Flytzani-Stephanopoulos, M. Low-temperature water-gas shift reaction over Cu- and Ni-loaded cerium oxide catalysts. *Appl. Catal. B* **27,** 179–191 (2000).
7. Yan, H. et al. Promoted Cu-Fe_3O_4 catalysts for low-temperature water gas shift reaction: optimization of Cu content. *Appl. Catal. B* **226,** 182–193 (2018).
8. Lin, J. et al. Remarkable performance of Ir_1/FeO_x single-atom catalyst in water gas shift reaction. *J. Am. Chem. Soc.* **135,** 15314−15317 (2013).
9. Panagiotopoulou, P., Kondarides, D. I. Effects of alkali promotion of TiO_2 on the chemisorptive properties and water–gas shift activity of supported noble metal catalysts. *J. Catal.* **267,** 57−66 (2009).
10. Yao, S. et al. Atomic-layered Au clusters on α-MoC as catalysts for the low-temperature water-gas shift reaction. *Science* **357,** 389–393 (2017).
11. Zhang, Z.-S. et al. Intrinsically active surface in a Pt/γ-Mo_2N catalyst for the water−gas shift reaction: molybdenum nitride or molybdenum oxide? *J. Am. Chem. Soc.* **142,** 13362–13371 (2020).
